# Early Metabolic Measures Predict Long-term Insulin Independence in Recipients of Total Pancreatectomy and Islet Autotransplantation

**DOI:** 10.1097/TXD.0000000000001561

**Published:** 2023-12-12

**Authors:** Yoshihide Nanno, James S. Hodges, Martin L. Freeman, Guru Trikudanathan, Sarah J. Schwarzenberg, Elissa M. Downs, Karthik Ramanathan, Timothy L. Pruett, Gregory J. Beilman, Srinath Chinnakotla, Bernhard J. Hering, Melena D. Bellin

**Affiliations:** 1 Department of Surgery, University of Minnesota, Minneapolis, MN.; 2 Schulze Diabetes Institute, Department of Surgery, University of Minnesota School of Medicine, Minneapolis, MN.; 3 Division of Hepato-Biliary-Pancreatic Surgery, Department of Surgery, Kobe University Graduate School of Medicine, Kobe, Japan.; 4 Division of Biostatistics, University of Minnesota, Minneapolis, MN.; 5 Department of Medicine, University of Minnesota, Minneapolis, MN.; 6 Department of Pediatrics, University of Minnesota, Minneapolis, MN.

## Abstract

**Background.:**

Although diabetes after total pancreatectomy and islet autotransplantation (TP-IAT) is one of the biggest concerns for TP-IAT recipients and physicians, reliable prediction of post-TP-IAT glycemic control remains unestablished. This study was conducted to identify early predictors of insulin independence and goal glycemic control by hemoglobin A1c (HbA1c) ≤ 6.5% after TP-IAT.

**Methods.:**

In this single-center, retrospective study, patients who underwent TP-IAT (n = 227) were reviewed for simple metabolic markers or surrogate indices of β-cell function obtained 3 mo after TP-IAT as part of standard clinical testing. Long-term metabolic success was defined as (1) insulin independence and (2) HbA1c ≤ 6.5% 1, 3, and 5 y after TP-IAT. Single- and multivariate modeling used 3-mo markers to predict successful outcomes.

**Results.:**

Of the 227 recipients, median age 31 y, 30% male, 1 y after TP-IAT insulin independence, and HbA1c ≤ 6.5% were present in 39.6% and 72.5%, respectively. In single-predictor analyses, most of the metabolic markers successfully discriminated between those attaining and not attaining metabolic goals. Using the best model selected by random forests analysis, we accurately predicted 1-y insulin independence and goal HbA1c control in 77.3% and 86.4% of the patients, respectively. A simpler “clinically feasible” model using only transplanted islet dose and BETA-2 score allowed easier prediction at a small accuracy loss (74.1% and 82.9%, respectively).

**Conclusions.:**

Metabolic testing measures performed 3 mo after TP-IAT were highly associated with later diabetes outcomes and provided a reliable prediction model, giving valuable prognostic insight early after TP-IAT and help to identify recipients who require early intervention.

Chronic pancreatitis is a progressive fibroinflammatory disease in which recurrent episodes of pancreatitis lead to irreversible fibrotic tissue replacement, resulting in intractable chronic pain and reduced quality of life.^1^ Total pancreatectomy and islet autotransplantation (TP-IAT) is a therapeutic option for chronic pancreatitis that is refractory to endoscopic and medical management, offering total removal of the pancreas to alleviate the pain while simultaneously preserving endogenous insulin secretion by transplanting the isolated islets back to the patient.^2,3^ Since the first case in 1977,^4^ TP-IAT has been offered worldwide in >1500 cases.^5^ Centers performing TP-IAT have individually reported that TP-IAT can relieve pain and restore quality of life in approximately 80% of recipients as well as preserve endogenous islet function in up to 90% of the patients and achieve insulin independence in 30%–50% of this patient population.^6-10^

Although a prospective observational multicenter study is currently underway to evaluate patient selection and timing for TP-IAT to further optimize outcomes,^11^ reliable prediction of post-TP-IAT glycemic control remains unestablished. In our previous study of predicting post-TP-IAT diabetes outcomes from pretransplant metabolic measures, the error rates for diabetes outcomes were as high as 30% even with the best statistically selected models.^12^ If better prediction of post-TP-IAT diabetes outcomes is possible from perioperative or early postoperative measures, it would benefit physicians and recipients in determining optimal targets for postoperative management.

The transplanted islet dose expressed in islet equivalents (IEQs) is an important prognostic factor for metabolic outcomes.^13-15^ It has been reported that an islet dose >4000–5000 IEQs/kg provides the best chance for insulin independence after TP-IAT.^8,16^ However, there is still much overlap between outcomes by islet dose.^12^ Many perioperative factors, such as islet graft loss after transplantation,^17-20^ variability in β-cell function,^21,22^ and recipient insulin sensitivity,^23^ are known to be responsible for this uncertainty. We commonly perform a metabolic stimulation test, a mixed meal tolerance test (MMTT), after TP-IAT to determine functional β-cell mass. MMTT is preferred to the oral glucose tolerance test^24,25^ as it produces less hyperglycemic stress on the transplanted islets, although available data on the relationship between post-TP-IAT metabolic measures obtained by MMTT and posttransplant glycemic control are limited.^14^ In this study, we evaluate the association of metabolic data early (3 mo) after transplant with long-term insulin independence and goal hemoglobin A1c (HbA1c) control (HbA1c ≤6.5%) in a large, well-phenotyped cohort of TP-IAT recipients. We aim to establish easy and reliable statistical models of insulin independence and goal HbA1c control after TP-IAT, which may contribute to patients and physicians by providing better prognostic insight early after TP-IAT.

## MATERIALS AND METHODS

### Study Subjects

We reviewed 419 consecutive TP-IAT cases at the University of Minnesota between October 2009 and December 2018 and identified 371 potentially eligible recipients after excluding those with partial pancreatectomy or presurgical diabetes, without research consent, or who did not complete 1-y follow-up. From this cohort, 227 recipients (159 adults age ≥18 y old and 68 children <18 y old) who had both complete laboratory data and daily insulin dose documented 3 mo after TP-IAT were included in the analyses (**Figure S1**, **SDC**, http://links.lww.com/TXD/A594). The study protocol was approved by the University of Minnesota Institutional Review Board (IRB No. 0609M91887). Informed consent, or parental permission and patient assent, were obtained from all participants as age appropriate.

### Total Pancreatectomy and Islet Autotransplantation

TP-IAT was performed according to previously published protocols.^8,12^ Briefly, the decision to proceed with TP-IAT was made on clinical grounds by a multidisciplinary team. On surgical excision of the pancreas, the blood supply to the pancreas was maintained until pancreatic mobilization was completed to minimize warm ischemia time and maximize islet preservation. After recovery, the pancreas was immediately transported to the University of Minnesota Molecular and Cellular Therapeutics good manufacturing practice facility. Islet isolation was performed using enzymatic digestion and mechanical dispersion as previously described.^26^ After digestion, the islets were purified using density gradients in a COBE 2991 cell processor (Terumo BCT, Lakewood, CO) if needed to reduce transplanted tissue volume (generally performed for a postdigest tissue volume >0.25 mL/kg).^27^ The final islet tissue preparation was transplanted by intraportal infusion during a 15- to 60-min period. In all cases, all or a majority of islets were transplanted intraportally, and a small proportion of islets were transplanted elsewhere (peritoneum, omentum) if portal pressures were elevated (typically above ~25 cm water).

### Postoperative Follow-up

Patients were seen postoperatively in outpatient clinics at 3 mo, 6 mo, 1 y, and annually thereafter. Daily insulin requirement doses were assessed during outpatient clinic visits and collected from self-reported survey data. Recipients are usually maintained on insulin for the first 3–6 mo after surgery to relieve β-cell functional stress during engraftment (neovascularization).^28^ Recipients are then weaned off insulin slowly as tolerated if they maintain these glycemic goals: HbA1c ≤6.5%, fasting blood glucose <126 mg/dL, and 2-h postprandial blood glucose level <180 mg/dL. Insulin therapy was restarted if recipients failed to meet these targets off treatment. Laboratory tests including MMTTs and HbA1c levels were performed as described below.^29^

### Metabolic Assessments

Recipients underwent MMTT 3 mo, 6 mo, and 1 y after TP-IAT.^30^ In MMTT, a patient consumed 6 mL/kg Boost high protein to a maximum of 360 mL >5 min at time 0, and glucose and C-peptide levels were measured at −1, +60, and +120 min. HbA1c levels were also obtained 3 mo, 6 mo, and 1 y after TP-IAT. The insulin dose was collected from both medical records and patient questionnaires (self-reported average daily use).

The following surrogate indices for β-cell function and/or insulin sensitivity were calculated using postoperative metabolic testing 3 mo after TP-IAT (Table 1): the Beta-score,^31^ BETA-2 score,^32^ C-peptide/glucose ratio (CP/G),^33^ Secretory Unit of Islets in Transplantation (SUITO) index,^34^ the Homeostasis Model Assessment (HOMA) estimating steady-state β-cell function (%B), insulin sensitivity (%S), and insulin resistance (IR), as percentages of a normal population.^35^ An insulin dose–adjusted HbA1c level (IDAA1c) was calculated from a previously described equation: HbA1c (%) + (4 × daily insulin dose [U]).^36^

**TABLE 1.** Surrogate indices of β-cell function and insulin sensitivity

Index	Characteristics			
Beta-score	Estimate graft function as a score between 8 (full β-cell function) and 0 (absolute absence of β-cell function)			
		Score of 2	Score of 1	Score of 0
	Fasting blood glucose (mmol/L)	≤5.5	5.6–6.9	≥7.0
	HbA1c (%)	≤6.1	6.2–6.9	≥7.0
	Daily insulin (U/kg) or OHA use	None	0.01–0.24 or OHA use	≥0.25
	Stimulated C-peptide (nmol/L)	≥0.3	0.1–0.29	<0.1
BETA-2 score	Estimate graft function as a continuous measure $\frac{\sqrt{fasting\;\;\;Cpeptide\;\;\;\left(nmol/L\right)}\;\;\;\times \;\;\;\left(1-insulin\;\;\;dose\;\;\;\left[Unit/kg\right]\right)}{fasting\;\;\;blood\;\;\;glucose\;\;\;\left(mmol/L\right)\times HbA1c\;\;\;\left(\%\right)}\;\;\;\times 1000$			
CP/G ratio	Account for the dependence of C-peptide secretion on glucose concentration $\frac{fasting\;\;\;Cpeptide\;\;\;\left(ng/mL\right)}{fasting\;\;\;blood\;\;\;glucose\;\;\;\left(mg/dL\right)}\times 1000$			
SUITO index	Estimate graft function as % compared with a healthy person $\frac{fasting\;\;\;Cpeptide\;\;\;\left(ng/mL\right)}{fasting\;\;\;blood\;\;\;glucose\;\;\;-63\;\;\;\left(ng/dL\right)}\times 1500$			
HOMA2-%B, %S, and IR	Calculated with the use of a computer program (HOMA calculator) (https://www.dtu.ox.ac.uk/homacalculator/download.php)			

CP/G, C-peptide/glucose ratio; HbA1c, hemoglobin A1C; HOMA2-%B, %S, and IR, Homeostasis Model Assessment 2 estimating steady-state β-cell function, insulin sensitivity, and insulin resistance; OHA, oral hypoglycemia agent; SUITO, Secretory Unit of Islets in Transplantation.

### Statistical Analysis

Patient characteristics are described as count (%) for categorical characteristics or median (interquartile range [IQR]) for continuous characteristics. To estimate and test simple associations between single 3-mo patient characteristics and diabetes outcomes 1, 3, or 5 y after TP-IAT, we used logistic regression with likelihood-ratio tests and confidence intervals computed from the likelihood. For multiple-predictor analyses, we used random forests to select predictors and logistic regression to obtain more interpretable prediction models. Specifically, for a given diabetes outcome after TP-IAT, all predictors with *P* < 0.05 in the random forests analyses were included in a logistic regression model to predict the diabetes outcome. Random forests analysis was performed using the randomForest package (v. 4.7-1.1) in the R system (v. 4.1.2) with 5000 trees, using 10 randomly selected predictors at each tree node; error rates and errors were obtained from out-of-bag predictions. *P* values were computed using the rfPermute package (version 2.5.1) using 1000 trees, 200 permutations, and otherwise the same settings; the minimum possible *P*-value was thus 0.00497. The logistic regression models were fit using the “glm” function in the base R package; cross-validated error rates, sensitivity, and specificity were computed using the “train” and “trainControl” functions in the caret package (v. 6.0-93).

## RESULTS

### Recipient Demographics

Table 2 shows the pretransplant demographics of the 227 TP-IAT recipients, which included recipients aged 3–67 y (30% [n = 68] aged <18 y). The primary cause of pancreatitis was most often genetic (41%), with the other most common etiologies being idiopathic (26%), pancreas divisum (17%), and Sphincter of Oddi dysfunction (11%). The median transplanted islet yield was 250 600 IEQs (IQR, 168 100–360 300 IEQs) and 4118 IEQs/kg (2809–6126 IEQs/kg). One hundred eighty-three (81%) recipients were transplanted total islet volume intraportally, and 44 (19%) recipients had both intraportal and extrahepatic transplantation.

**TABLE 2.** Recipients’ pretransplant demographics and diabetes outcomes

Characteristic	Analyzed cohort (n = 227)
Age, y, median (IQR)	31 (16–45)
Age < 18, n (%)	68 (30)
Male sex, n (%)	69 (30)
Pretransplant BMI, kg/m^2^, median (IQR)	23 (19.8–27.6)
Duration of diagnosed pancreatitis, y, median (IQR)	4.4 (2.2–8.5)
Etiology of chronic pancreatitis, n (%)	
Hereditary disease^*a*^^,^^*b*^	92 (41)
Pancreas divisum^*a*^	39 (17)
Sphincter of Oddi dysfunction^*b*^	25 (11)
Alcohol	4 (2)
Others	10 (4)
Idiopathic	59 (26)
Previous pancreas surgery, n (%)	17 (7)
Preoperative HbA1c level,^*c*^ %, median (IQR)	5.3 (5.1–5.5)
Preoperative glucose level, mg/dL, median (IQR)	
Fasting^*c*^	88 (83–94)
1 h after MMTT^*d*^	89 (78–109)
2 h after MMTT^*d*^	90 (82–101)
Preoperative C-peptide level, ng/mL, median (IQR)	
Fasting^*e*^	1.5 (1.1–2.1)
Stimulated^*f*^	5.2 (3.6–7.4)
Estimated pancreas volume,^*g*^ cm^3^/kg, median (IQR)	0.390 (0.289–0.524)
Pancreas weight, g/kg, median (IQR)	1.02 (0.80–1.38)
Extent of fibrosis, n (%)	
Mild (1–3)	34 (15)
Intermediate (4–7)	87 (38)
Severe (8–10)	106 (47)
Transplanted islet mass, IEQ/kg, median (IQR)	4118 (2809–6126)
Transplanted islet number, IPN/kg, median (IQR)	4780 (3307–6816)
With COBE procedure, n (%)	69 (30)
Beta-score,^*h*^ median (IQR)	8 (7–8)
BETA-2 score,^*i*^ median (IQR)	28.2 (22.4–33.0)
SUITO index,^*j*^ median (IQR)	97.8 (61.4–136.4)
CP/G ratio,^*k*^ median (IQR)	17.3 (11.7–24.1)
HOMA2-%B,^*k*^ median (IQR)	110.3 (79.7–137.5)
HOMA2-%S,^*k*^ median (IQR)	92.5 (65.6–127.3)
HOMA2-IR,^*k*^ median (IQR)	1.1 (0.8–1.5)
Insulin independence at 1 y after TP-IAT, n (%)	90 (39.6)
HbA1c ≤ 6.5% at 1 y after TP-IAT,^*l*^ n (%)	161 (72.5)
Severe hypoglycemic events within 1 y after TP-IAT,^*m*^ n (%)	9 (4.1)

^*a*^ Four patients had both pancreas divisum and hereditary background.

^*b*^ One patient had both sphincter of Oddi dysfunction and hereditary background.

^*c*^ 370 patients were evaluated.

^*d*^ 355 patients were evaluated.

^*e*^ 369 patients were evaluated.

^*f*^ 358 patients were evaluated.

^*g*^ 250 patients were evaluated.

^*h*^ 364 patients were evaluated.

^*i*^ 367 patients were evaluated.

^*j*^ 366 patients were evaluated.

^*k*^ 368 patients were evaluated.

^*l*^ 346 patients were evaluated.

^*m*^ 348 patients were evaluated.

BMI, body mass index; CP/G, C-peptide/glucose ratio; HbA1c, hemoglobin A1C; HOMA2-%B, Homeostasis Model Assessment 2 estimating steady-state β-cell function; HOMA2-%S, Homeostasis Model Assessment 2 insulin sensitivity; HOMA2-IR, Homeostasis Model Assessment 2 insulin resistance; IEQ, islet equivalent; IPN, islet particle number; IQR, interquartile range; MMTT, mixed meal tolerance test; SUITO, Secretory Unit of Islets in Transplantation; TP-IAT, total pancreatectomy and islet autotransplantation.

Ninety (39.6%) recipients were insulin independent 1 y after TP-IAT. HbA1c levels 1 y after TP-IAT were available for 222 recipients; the median HbA1c level was 6.4% (IQR, 5.7%–7.5%), and 161 (72.5%) recipients achieved goal glycemic control (HbA1c ≤ 6.5%). Sufficient data to determine incidence of severe hypoglycemic events were obtained for 218 recipients, with events reported in 9 (4.1%) recipients within 1 y after TP-IAT.

### Relationship Between Recipient Characteristics 3 mo After TP-IAT and Diabetes Outcomes 1 y After TP-IAT

Table 3 shows results of single-predictor analyses of the association between patient characteristics 3 mo after TP-IAT and insulin independence 1, 3, and 5 y after TP-IAT. Higher transplanted islet dose, lower HbA1c and IDAA1c levels, lower daily insulin dose, and lower fasting and stimulated blood glucose levels from MMTT at 3 mo were significantly associated with increased odds of insulin independence 1, 3, and 5 y after TP-IAT. Higher stimulated C-peptide level was marginally significantly associated with increased odds of insulin independence 1 and 3 y and significantly associated 5 y after TP-IAT. Fasting C-peptide level was not significantly associated with insulin independence 1 and 3 y but significantly associated with increased odds of insulin independence 5 y after TP-IAT, suggesting robust insulin production is more predictive of sustaining insulin independence long-term.

**TABLE 3.** Single-predictor logistic regression analyses for association between recipient characteristics 3 mo after TP-IAT and insulin independence 1, 3, and 5 y after TP-IAT

Variables	Mean (SD) or n (%)	1 y (n = 227)		3 y (n = 194)		5 y (n = 130)	
		OR (95% CI)	*P*	OR (95% CI)	*P*	OR (95% CI)	*P*
Age, y	30.9 (16.5)	0.91 (0.69-1.19)	0.48	0.80 (0.60-1.05)	0.082	0.68 (0.47-0.97)	0.034
Sex (female)	158 (69.6%)	1.61 (0.90-2.97)	0.11	1.81 (0.97-3.43)	0.060	0.70 (0.32-1.50)	0.36
Transplanted islet dose, IEQ/kg	4607 (2494)	1.51 (1.32-1.76)	**< 0.001**	1.78 (1.50-2.16)	**< 0.001**	1.69 (1.39-2.10)	**< 0.001**
BMI (adults only), kg/m^2^	23.9 (4.2)	0.73 (0.51-1.04)	0.084	0.82 (0.56-1.19)	0.29	0.88 (0.56-1.35)	0.57
HbA1c level, %	6.1 (0.7)	0.17 (0.09-0.29)	**< 0.001**	0.18 (0.09-0.31)	**< 0.001**	0.22 (0.10-0.41)	**< 0.001**
IDAA1c level, %	7.0 (1.3)	0.06 (0.03-0.13)	**< 0.001**	0.12 (0.06-0.23)	**< 0.001**	0.23 (0.11-0.43)	**< 0.001**
Daily insulin dose, U/kg	0.225 (0.209)	0.10 (0.04-0.19)	**< 0.001**	0.20 (0.10-0.36)	**< 0.001**	0.38 (0.19-0.68)	**< 0.001**
Fasting blood glucose level, mg/dL	103 (29)	0.32 (0.19-0.52)	**< 0.001**	0.31 (0.17-0.52)	**< 0.001**	0.40 (0.20-0.72)	**0.001**
Stimulated (1-h) blood glucose level, mg/dL	142 (53)	0.17 (0.09-0.29)	**< 0.001**	0.20 (0.11-0.34)	**< 0.001**	0.21 (0.10-0.40)	**< 0.001**
Stimulated (2-h) blood glucose level, mg/dL	130 (57)	0.10 (0.04-0.20)	**< 0.001**	0.13 (0.06-0.26)	**< 0.001**	0.15 (0.05-0.35)	**< 0.001**
Fasting C-peptide level, ng/mL	0.92 (0.60)	1.08 (0.83-1.41)	0.57	1.34 (1.00-1.83)	0.052	1.46 (1.01-2.22)	0.047
Stimulated C-peptide level, ng/mL	2.53 (1.63)	1.37 (1.05-1.84)	0.021	1.37 (1.03-1.89)	.033	1.69 (1.14-2.69)	**0.007**
Beta-score	5.6	3.45 (2.46-5.08)	**< 0.001**	2.96 (2.14-4.29)	**< 0.001**	2.05 (1.47-3.01)	**< 0.001**
BETA-2 score	13.1 (6.5)	3.86 (2.62-5.96)	**< 0.001**	4.86 (3.05-8.25)	**< 0.001**	3.65 (2.21-6.51)	**< 0.001**
SUITO index	42.2 (29.1)	1.80 (1.34-2.50)	**< 0.001**	2.60 (1.76-4.06)	**< 0.001**	2.59 (1.60-4.52)	**< 0.001**
C-peptide/glucose ratio	9.2 (5.8)	1.06 (1.01-1.11)	0.024	1.10 (1.04-1.17)	**< 0.001**	1.12 (1.05-1.22)	**< 0.001**
HOMA2-%B	59.4 (29.8)	2.09 (1.52-2.96)	**< 0.001**	2.72 (1.85-4.19)	**< 0.001**	2.64 (1.65-4.50)	**< 0.001**
HOMA2-%S	258 (285)	0.89 (0.80-0.97)	**0.008**	0.84 (0.73-0.94)	**0.002**	0.81 (0.65-0.95)	**0.006**
HOMA2-IR	0.70 (0.47)	1.00 (0.76-1.31)	0.99	1.23 (0.92-1.67)	0.16	1.32 (0.92-1.97)	0.14

Significant *P* (< 0.01) are in bold. Marginally significant *P* (< 0.05) are underlined.

ORs and 95% CIs are calculated for a 1 SD increase in the measure with these exceptions: the rows for sex, transplanted islet dose, Beta-score, and HOMA2-%S show the OR associated with, respectively, being male, a 1000 IEQ/kg increase, a 1-unit increase, and an increase of 1/2 of the interquartile range.

BMI, body mass index; CI, confidence interval; HbA1c, hemoglobin A1C; HOMA2-%B, Homeostasis Model Assessment 2 estimating steady-state β-cell function; HOMA2-%S, Homeostasis Model Assessment 2 insulin sensitivity; HOMA2-IR, Homeostasis Model Assessment 2 insulin resistance; IDAA1c, insulin dose–adjusted HbA1c; IEQ, islet equivalent; OR, odds ratio; SUITO, Secretory Unit of Islets in Transplantation; TP-IAT, total pancreatectomy and islet autotransplantation.

Single-predictor analyses of the association between metabolic measures and goal HbA1c control also found that 3-mo HbA1c and IDAA1c levels, daily insulin dose, and fasting and stimulated blood glucose levels were negatively associated with HbA1c ≤6.5% 1, 3, and 5 y after TP-IAT (Table 4). Transplanted islet dose and fasting and stimulated C-peptide levels were positively associated with goal HbA1c control 1, 3, and 5 y after TP-IAT. Females were more likely to attain goal HbA1c control 1 y after TP-IAT, but this was not significant at 5 y. Age was not significantly associated with goal HbA1c control 1 y after TP-IAT but was marginally significantly associated 5 y after TP-IAT. These associations showed the same directionality as those for insulin independence.

**TABLE 4.** Single-predictor logistic regression analyses for association between recipient characteristics 3 mo after TP-IAT and goal HbA1c control (≤6.5%) 1, 3, and 5 y after TP-IAT

Variables	Mean (SD) or n (%)	1 y (n = 222)		3 y (n = 180)		5 y (n = 129)	
		OR (95% CI)	*P*	OR (95% CI)	*P*	OR (95% CI)	*P*
Age, y	30.9 (16.5)	0.81 (0.60-1.09)	0.17	0.74 (0.55-0.98)	0.038	0.65 (0.44-0.94)	0.022
Sex (female)	158 (69.6%)	2.83 (1.53-5.28)	**0.001**	2.29 (1.19-4.45)	0.013	1.08 (0.48-2.35)	0.86
Transplanted islet dose, IEQ/kg	4607 (2494)	1.57 (1.32-1.90)	**< 0.001**	1.53 (1.30-1.83)	**< 0.001**	1.31 (1.11-1.58)	**0.001**
BMI (adults only), kg/m^2^	23.9 (4.2)	0.78 (0.54-1.11)	0.17	0.92 (0.63-1.34)	0.64	0.91 (0.60-1.41)	0.68
HbA1c level, %	6.1 (0.7)	0.09 (0.04-0.18)	**< 0.001**	0.27 (0.15-0.44)	**< 0.001**	0.45 (0.25-0.77)	**0.003**
IDAA1c level, %	7.0 (1.3)	0.17 (0.10-0.28)	**< 0.001**	0.27 (0.16-0.43)	**< 0.001**	0.36 (0.19-0.63)	**< 0.001**
Daily insulin dose, U/kg	0.225 (0.209)	0.33 (0.21-0.49)	**< 0.001**	0.37 (0.23-0.57)	**< 0.001**	0.41 (0.22-0.73)	**0.002**
Fasting blood glucose level, mg/dL	103 (29)	0.31 (0.19-0.48)	**< 0.001**	0.36 (0.20-0.58)	**< 0.001**	0.65 (0.36-1.09)	0.10
Stimulated (1-h) blood glucose level, mg/dL	142 (53)	0.21 (0.13-0.32)	**< 0.001**	0.24 (0.14-0.38)	**< 0.001**	0.42 (0.25-0.68)	**< 0.001**
Stimulated (2-h) blood glucose level, mg/dL	130 (57)	0.17 (0.09-0.28)	**< 0.001**	0.18 (0.09-0.32)	**< 0.001**	0.25 (0.12-0.47)	**< 0.001**
Fasting C-peptide level, ng/mL	0.92 (0.60)	1.92 (1.31-2.92)	**< 0.001**	1.67 (1.18-2.45)	**0.003**	2.24 (1.38-3.93)	**< 0.001**
Stimulated C-peptide level, ng/mL	2.53 (1.63)	2.44 (1.57-4.04)	**< 0.001**	1.92 (1.31-2.98)	**< 0.001**	2.13 (1.29-3.91)	**0.002**
Beta-score	5.6	2.71 (2.05-3.74)	**< 0.001**	2.01 (1.55-2.69)	**< 0.001**	1.41 (1.06-1.93)	0.019
BETA-2 score	13.1 (6.5)	8.39 (4.76-16.4)	**< 0.001**	3.97 (2.53-6.63)	**< 0.001**	3.72 (2.13-7.11)	**< 0.001**
SUITO index	42.2 (29.1)	8.82 (4.57-18.8)	**< 0.001**	4.33 (2.55-7.90)	**< 0.001**	3.03 (1.70-5.98)	**< 0.001**
C-peptide/glucose ratio	9.2 (5.8)	1.23 (1.14-1.34)	**< 0.001**	1.15 (1.08-1.25)	**< 0.001**	1.18 (1.08-1.31)	**< 0.001**
HOMA2-%B	59.4 (29.8)	7.09 (4.07-13.4)	**< 0.001**	3.4 (2.17-5.62)	**< 0.001**	2.76 (1.65-5.01)	**< 0.001**
HOMA2-%S	258 (285)	0.88 (0.81-0.95)	**< 0.001**	0.87 (0.77-0.97)	0.01	0.82 (0.68-0.94)	**0.002**
HOMA2-IR	0.70 (0.47)	1.61 (1.13-2.39)	**0.008**	1.49 (1.07-2.16)	0.018	2.08 (1.30-3.62)	**0.002**

Significant *P* (< 0.01) are in bold. Marginally significant *P* (< 0.05) are underlined.

ORs and 95% CIs are calculated for a 1 SD increase in the measure with these exceptions: the row for sex, transplanted islet dose, Beta-score, and HOMA2-%S show, respectively, the OR associated with being male, a 1000 IEQ/kg increase, a 1-unit increase, and an increase of 1/2 of the interquartile range change.

BMI, body mass index; CI, confidence interval; HbA1c, hemoglobin A1C; HOMA2-%B, Homeostasis Model Assessment 2 estimating steady-state β-cell function; HOMA2-%S, Homeostasis Model Assessment 2 insulin sensitivity; HOMA2-IR, Homeostasis Model Assessment 2 insulin resistance; IDAA1c, insulin dose–adjusted HbA1c; IEQ, islet equivalent; OR, odds ratio; SUITO, Secretory Unit of Islets in Transplantation; TP-IAT, total pancreatectomy and islet autotransplantation.

### Relationship Between Surrogate Indices 3 mo After TP-IAT and Diabetes Outcomes 1 y After TP-IAT

Single-predictor analyses of the association between surrogate indices and insulin independence found that the odds of insulin independence increased >2-fold 1, 3, and 5 y for each 1-unit increase in the 3-mo Beta-score (Table 3). A 1 SD increase in the BETA-2 score also increased the odds for insulin independence by >3.5-fold 1, 3, and 5 y after TP-IAT. The SUITO index and HOMA2-%B were also associated with insulin independence 1, 3, and 5 y after TP-IAT. CP/G was marginally associated with insulin independence 1 y after TP-IAT but was significantly associated with 3 and 5 y. In contrast, a 1 SD increase in HOMA2-%S reduced the odds for insulin independence 1, 3, and 5 y by about 0.8–0.9-fold, and HOMA2-IR showed no association.

Single-predictor analyses of the association between surrogate indices and goal HbA1c control showed that the Beta-score, BETA-2 score, SUITO index, CP/G, and HOMA2-%B, and HOMA2-IR were positively associated with achieving goal HbA1c control 1, 3, and 5 y after TP-IAT (Table 4). HOMA2-%S was negatively associated with achieving goal HbA1c control 1, 3, and 5 y after TP-IAT.

### Multiple-predictor Analyses for Diabetes Outcomes and Patient Predictions

**FIGURE 1.**
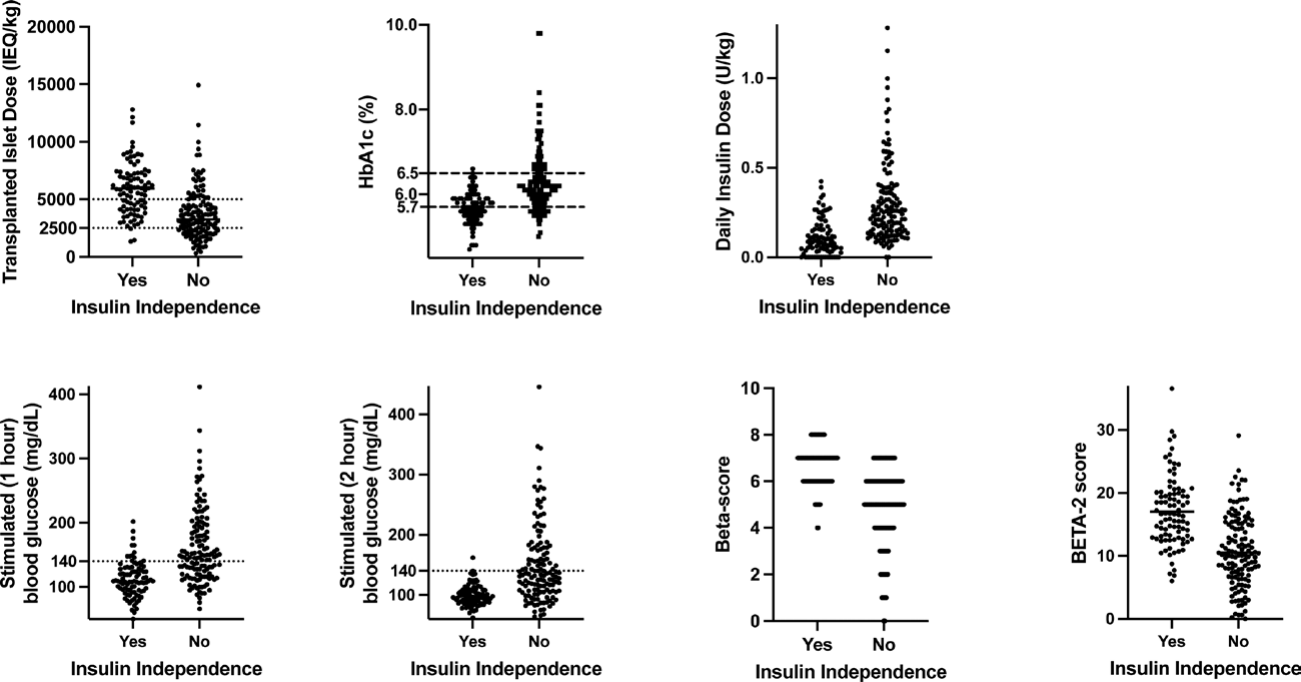
Association between patient characteristics and insulin independence 1 y after TP-IAT. HbA1c, hemoglobin A1C; IEQ, islet equivalent; TP-IAT, total pancreatectomy and islet autotransplantation.

**FIGURE 2.**
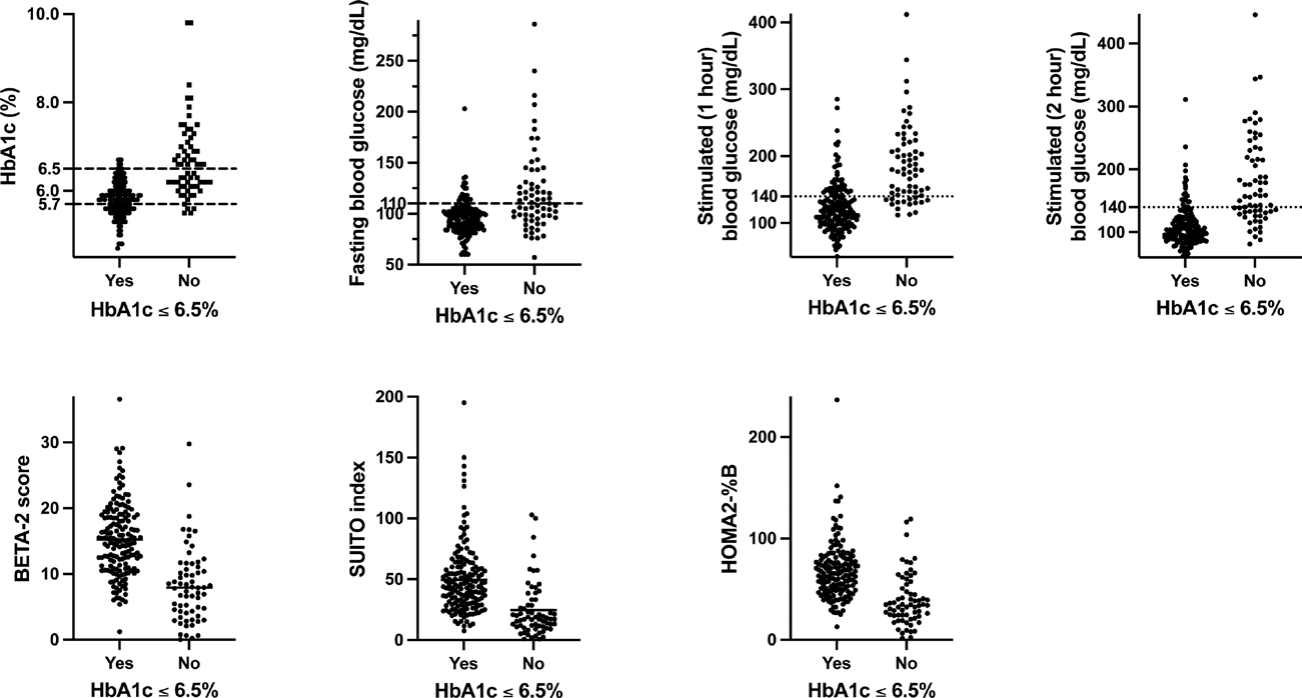
Association between patient characteristics and attaining goal (≤6.5%) HbA1c control 1 y after TP-IAT. HbA1c, hemoglobin A1C; HOMA2-%B, Homeostasis Model Assessment 2 estimating steady-state β-cell function; SUITO, Secretory Unit of Islets in Transplantation; TP-IAT, total pancreatectomy and islet autotransplantation.

**FIGURE 3.**
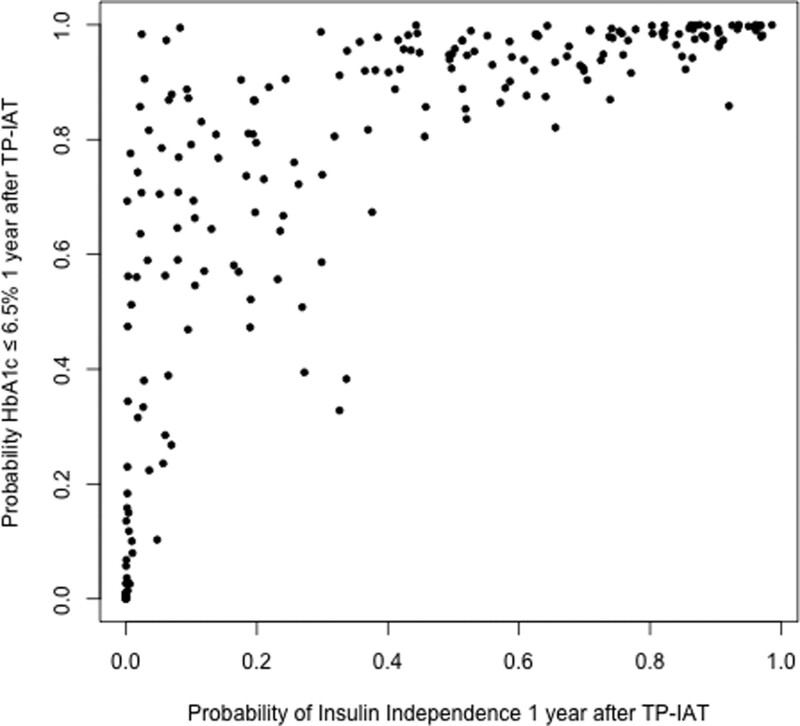
Scatter plot that shows predicted probability of achieving goal (≤6.5%) HbA1c control against predicted probability of insulin independence 1 y after TP-IAT. The 2 predictions show fair correlation with each other (*r* = 0.73; 95% confidence interval, 0.62-0.79; *P* < .0001). HbA1c, hemoglobin A1C; TP-IAT, total pancreatectomy and islet autotransplantation.

To construct multivariate models, we first included age, sex, IEQ/kg, insulin dose, and all measured and calculated metabolic measures (except IDAA1c) in a random forests analysis. Statistically significant variables (*P* < 0.05, Table 5) from the random forests analyses are shown in Figures 1 and 2. Using these variables, we estimated logistic regression equations for 1 y insulin independence and goal HbA1c control (**Supplemental Information**, **SDC**, http://links.lww.com/TXD/A594); they correctly predicted insulin independence and goal HbA1c control (by cross-validation) 77.3% and 86.4% of the time, respectively (Table 6). The predicted probability of insulin independence and goal HbA1c control showed a strong correlation with each other (Figure 3).

**TABLE 5.** *P* from the random forests analyses using recipient characteristics 3 mo after TP-IAT to predict insulin independence and goal HbA1c control (≤6.5%) 1, 3, and 5 y after TP-IAT

Variables	Insulin independence			HbA1c ≤ 6.5%		
	At 1 y	At 3 y	At 5 y	At 1 y	At 3 y	At 5 y
Age, y	0.0597	0.3233	**0.0497**	0.3830	0.5472	**0.0398**
Sex	0.6766	0.5820	0.4228	0.4278	0.1592	0.3980
Transplanted islet dose, IEQ/kg	**0.0099**	**0.0049**	**0.0049**	0.3781	**0.0099**	0.1293
HbA1c level, %	**0.0149**	**0.0199**	**0.0248**	**0.0049**	0.1044	0.1393
Daily insulin dose, U/kg	**0.0049**	**0.0099**	0.1243	0.1691	0.2089	0.1243
Fasting blood glucose level, mg/dL	0.2238	0.1044	0.2985	**0.0497**	**0.0298**	0.0646
Stimulated (1-h) blood glucose level, mg/dL	**0.0049**	**0.0099**	**0.0049**	**0.0099**	**0.0049**	0.1542
Stimulated (2-h) blood glucose level, mg/dL	**0.0049**	**0.0049**	0**.0398**	**0.0099**	**0.0049**	**0.0348**
Fasting C-peptide level, ng/mL	0.1542	0.3383	0.5273	0.2786	0.0696	0.0895
Stimulated C-peptide level, ng/mL	0.3880	0.1890	0.0796	0.1791	**0.0298**	0.1691
Beta-score	**0.0049**	**0.0298**	0.1442	0.2288	0.7114	0.1343
BETA-2 score	**0.0049**	**0.0049**	**0.0398**	**0.0049**	**0.0099**	**0.0199**
SUITO index	0.4975	0.1393	0.1791	**0.0099**	**0.0049**	**0.0049**
C-peptide/glucose ratio	0.2039	0.3084	0.2885	0.4427	0.1442	**0.0049**
HOMA2-%B	0.2537	**0.0298**	0.1393	**0.0248**	**0.0049**	**0.0149**
HOMA2-%S	0.1890	0.1940	0.4129	0.1393	0.0696	0.0895
HOMA2-IR	0.2786	0.2885	0.4776	0.0945	0.0845	0.0945

Significant *P* (< 0.05) are in bold. *P* < 0.10 are underlined.

HbA1c, hemoglobin A1C; HOMA2-%B, Homeostasis Model Assessment 2 estimating steady-state β-cell function; HOMA2-%S, Homeostasis Model Assessment 2 insulin sensitivity; HOMA2-IR, Homeostasis Model Assessment 2 insulin resistance; IEQ, islet equivalent; SUITO, Secretory Unit of Islets in Transplantation; TP-IAT, total pancreatectomy and islet autotransplantation.

**TABLE 6.** Error rates, sensitivity, and specificity for the logistic regression models^*a*^

Metabolic outcomes 1 y after TP-IAT	Using all predictors *P* < .05 in random forests analyses			Using only “transplanted islet dose” and “BETA-2 score”		
	Model error rate (%)	Sensitivity	Specificity	Model error rate (%)^*a*^	Sensitivity	Specificity
Insulin independence	22.7%	73%	80%	25.9%	60%	83%
HbA1c ≤6.5%	13.6%	95%	64%	17.1%	93%	59%

^*a*^ By cross-validation.

HbA1c, hemoglobin A1C; TP-IAT, total pancreatectomy and islet autotransplantation.

The statistically driven multivariate modeling produced models were strongly predictive but hard to implement in clinical practice. For this reason, we also constructed a simpler prediction model using 2 mutually exclusive variables, 3-mo BETA-2 and transplanted islet dose, to predict insulin independence and HbA1c at 1 y as described (**Supplemental Information**, **SDC**, http://links.lww.com/TXD/A594). BETA-2 score is an easy surrogate measure that requires only daily insulin dose and a blood sample in a fasting state; it is strongly associated with both insulin independence and goal HbA1c level. Transplanted islet dose was included as the most important surgical characteristic associated with transplant outcomes.^8^ The resulting simple equation correctly predicted insulin independence and goal HbA1c control (by cross-validation) 74.1% and 82.9% of the time, respectively (Table 5). Table 7 displays characteristics for 12 example patients selected for having a high, moderate, or low chance for insulin independence 1 y after TP-IAT, as predicted by the multivariate and simple models.

**TABLE 7.** Probability of insulin independence 1 y after TP-IAT from multivariate logistic regression model and simple equation model

Probability of insulin independence	Descriptors			Predictors							Prediction rate by best statistical model	Prediction rate by simple model	Insulin status at 1 y after TP-IAT
	Age	Sex	BMI	Transplanted islet dose, 1000 IEQ/kg	HbA1c, %	Daily insulin dose, U/kg	Stimulated (1 h) glucose, mg/dL	Stimulated (2 h) glucose, mg/dL	Beta-score	BETA-2 score			
High	39	Male	25.0	8769	5.3	0.027	93	100	7	21.73	95.0%	88.8%	Independent
	24	Female	37.2	5895	5.0	0.104	96	106	7	24.68	86.2%	85.4%	Independent
	41	Female	26.6	4895	5.4	0.072	76	109	7	18.97	86.0%	62.4%	Independent
	36	Female	29.4	2753	5.2	0.025	87	102	7	24.54	82.3%	70.3%	Independent
Med	15	Male	23.3	4225	5.8	0.174	116	99	7	15.16	53.2%	41.6%	Dependent
	54	Male	24.6	3812	5.5	0.108	104	134	6	20.14	52.7%	59.9%	Independent
	47	Female	20.6	5460	6.2	0.098	119	107	5	14.23	45.6%	46.3%	Independent
	52	Female	27.6	2992	5.6	0.156	132	120	7	14.39	43.5%	30.6%	Dependent
Low	49	Male	27.8	3876	6.1	0.079	166	129	5	8.53	26.9%	17.1%	Dependent
	53	Female	21.5	2485	6.8	0.127	145	132	4	11.58	6.0%	19.1%	Dependent
	13	Female	19.2	4105	6.1	0.361	172	107	5	9.24	6.0%	20.0%	Dependent
	21	Male	32.6	2076	6.8	0.242	147	133	5	16.79	2.4%	34.0%	Dependent

Twelve-patient examples are shown from a high, moderate, and low probability group to illustrate the relationship between the patient characteristics and probability of insulin independence.

BMI, body mass index; HbA1c, hemoglobin A1C; IEQ, islet equivalent; TP-IAT, total pancreatectomy and islet autotransplantation.

## DISCUSSION

At our institution, after TP-IAT all patients are treated with insulin during the early postoperative period to reduce metabolic stress during islet engraftment, and most patients achieving insulin independence are not fully weaned off insulin until 6 mo to 1 y or more after surgery. A common question from patients after TP-IAT is how likely they are to stop insulin therapy in the future. In this study, we used measures including islet yield, insulin dosing, and routinely obtained early (3 mo) laboratory data including glycemic control and C-peptide production to determine if early measures could predict later insulin independence and glycemic outcomes. Using the best statistically selected model, we could accurately predict 1 y insulin independence in 77.3% of patients at 3 mo after TP-IAT, with a sensitivity of 73% and a specificity of 80%. A simpler model using only transplanted islet dose and BETA-2, which can be calculated without a metabolic stimulation test, successfully predicted outcomes in 74.1% of persons with a sensitivity of 60% and a specificity of 83%. The simpler model may allow easier implementation in a clinical setting at the cost of a small loss in accuracy. These models should be used with awareness of their limitations but may give providers and patients better prognostic insight early after TP-IAT.

The primary goal of TP-IAT is to relieve pain and restore quality of life. As a trade-off, TP-IAT recipients must accept the risk of postoperative insulin-dependent diabetes and life-long commitment to pancreatic enzyme replacement therapy.^37^ All patients require insulin for several months after surgery. However, patients and families are eager for early prognostic markers of expected outcomes, especially regarding whether they are likely to stop insulin. Thus, our study provides new insights that can be used to provide prognostic counseling for patients early after TP-IAT. It may be stressed that our insights that BETA-2 is a key factor that predict metabolic outcome was compatible with the findings of a recent large-scale analysis (n = 255) of allogeneic islet transplantation from University of Alberta that revealed higher BETA-2 score was associated with better metabolic outcome.^38^

Exogenous insulin treatment during the early stage of engraftment improves the outcome of islet transplantation by minimizing the workload experienced by the transplanted grafts and is therefore considered to be of great importance.^39^ Previous experimental studies have also shown that islet cells are at a baseline disadvantage to sustain themselves during times of metabolic stress as they lack a robust antioxidant defense system,^40^ and future early interventions to reduce oxidative stress for these vulnerable recipients may better preserve transplanted islet grafts and improve the outcome of islet transplantation.^41^ While our study may not directly change early postoperative management of TP-IAT patients (which will continue to focus on tight glycemic control), it could identify a group of patients who have a higher risk for “poor” diabetes outcomes and who could thus be triaged earlier to more intensive insulin therapies such as closed-loop pumps, or who could be candidates for future studies of interventions to improve islet graft survival. In the future, as newer cell sources (like stem-cell derived islets) become available, these tests could be used to identify candidates for additional cellular therapy.

We found some similarities and dissimilarities between our current study and our previous study that predicted glycemic control from preoperative metabolic parameters.^14^ There is a substantial overlap in baseline measures between the groups who were insulin independent and dependent: recipients with abnormal glycemia (prediabetes HbA1c or fasting/stimulated blood glucose levels) at pretransplant or posttransplant assessment or recipients with low transplanted islet dose (<2500 IEQs/kg) appeared to have a very low likelihood of insulin independence.^12^ Conversely, higher posttransplant stimulated C-peptide levels and higher CP/G after surgery were associated with higher odds for insulin independence and glycemic control in the current and other previous studies,^30,33^ however, higher pretransplant stimulated C-peptide levels and higher CP/G were negatively associated with insulin independence and glycemic control after surgery.^12^ C-peptide levels were very different between pre- and posttransplant conditions: Mean stimulated C-peptide level was lower by 60% in the postoperative settings compared with the preoperative settings (2.5 versus 6.3 ng/mL). This seems consistent with the results of previous studies showing that 50%–70% of transplanted β cells are lost in the early postoperative period.^17,18^ The posttransplant C-peptide level and CP/G may reflect functional β-cell mass and the pretransplant C-peptide level and CP/G may reflect insulin resistance, which should be appraised when interpreting the results.

The MMTT is a metabolic stimulation test commonly performed after TP-IAT to monitor the function of transplanted islets.^42^ While early postoperative metabolic measures obtained from MMTT could statistically discriminate those who achieved insulin independence and goal HbA1c control from those who did not at a population level, there will still be an overlap between those who did and did not achieve these metabolic successes (Figures 1 and 2). The MMTT is a simple-to-administer physiological test that incorporates the incretin response, and the rate of nutrient absorption into systemic circulation may affect the test’s sensitivity.^24^ Also, recipients of TP-IAT have received reconstruction of the gastrointestinal tract and may have impaired secretion of the glucose-dependent insulinotropic polypeptide.^43^ More sensitive measures for detecting early deficits in β-cell function and function such as oral glucose tolerance test and glucose-potentiated arginine stimulation may help to improve prediction and could be incorporated into future studies; however, they may not be easily performed in a clinical setting.^44-46^ Continuous glucose monitoring (CGM) is now used routinely in our practice early after TP-IAT (which was not the case in 2009–2018, the timeframe of the current study). While CGM only provides data on glycemia and not insulin secretion, CGM does reflect a longer time frame of “real world” glycemia that may be incorporated into future studies.

This study has some limitations that should be noted. First, this retrospective study analyzed a subset of the total recipient population because the 1-y survey and early metabolic assessment were not completed for all recipients. This restriction may have created selection bias. Second, this was a single-center study with a specific strategy for patient selection and management and may lack external validity. Third, some metabolic measures could have influenced patient management; for example, patients with high body mass index or marginal blood glucose or C-peptide levels may have been preferentially maintained on insulin for longer than patients without concerns of metabolic stress on the islets.

In conclusion, our data showed that, by incorporating early metabolic measures, we could accurately predict insulin independence and goal HbA1c control after TP-IAT. The best statistically selected model predicted outcomes with 77.3% accuracy, and the simpler model using transplanted islet dose and BETA-2 predicted outcomes with 74.1% accuracy. Reliable prediction of metabolic outcomes provides targets for postoperative management and can help identify recipients who require early intervention to mitigate metabolic stress. Future research should investigate additional measures to enable more reliable postoperative assessment and management.

## ACKNOWLEDGMENTS

We gratefully acknowledge the islet isolation team members of Schulze Diabetes Institute, University Minnesota, for their dedicated work: Thomas Gilmore, Jeffrey D. Ansite, Joshua J. Wilhelm, Muhamad Abudulla, David Heller, and Zachary Swanson.

## Supplementary Material


