# Supplementary material for: Early Metabolic Measures Predict Long-term Insulin Independence in Recipients of Total Pancreatectomy and Islet Autotransplantation
Source: Transplant Direct. 2023 Dec 12;10(1):e1561. doi: 10.1097/TXD.0000000000001561 (PMC10715795; doi:10.1097/TXD.0000000000001561)
Supplement: Supplementary file 1 [file txd-10-e1561-s001.pdf]

SUPPLEMENTAL DIGITAL CONTENT

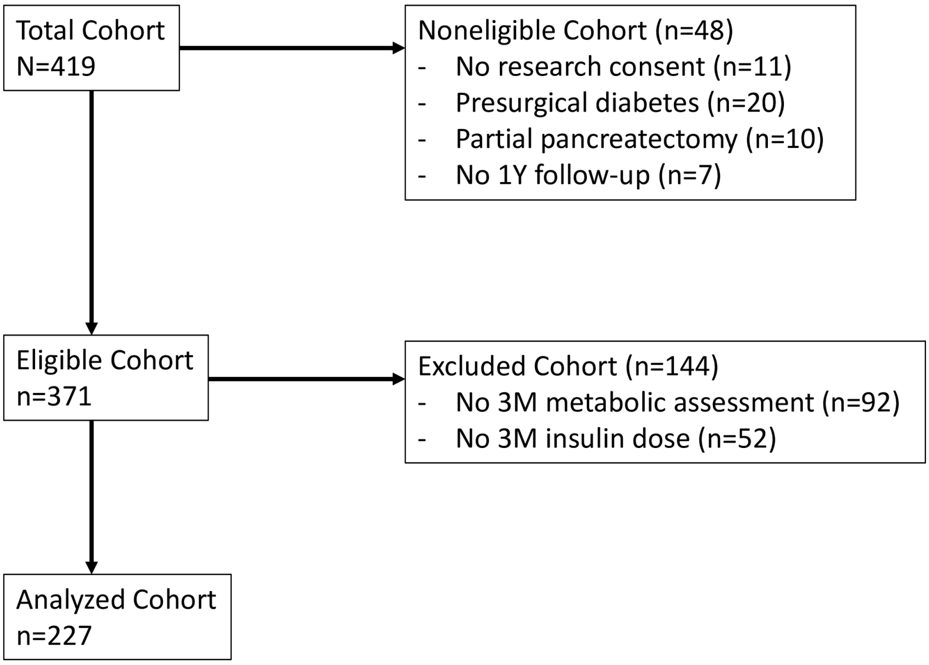

Supplementary Figure S1. A flow diagram of the study.

Supplemental Information

Below, π is the estimated probability of a good outcome and logit(π) = ln(π / (1-π)).

Prediction of Insulin Independence at 1 Year: Logistic regression formula using all predictors *P* < .05 in random forests analyses

In the formula below, π indicates the probability of being insulin independent 1 year after TP-IAT.

logit(π) = 10.1 + 0.248**Transplanted islet dose (1000 IEQ/kg)* – 1.34**HbA1c level 3 months after TP-IAT (%)* – 7.01**daily insulin dose 3 months after TP-IAT (U/kg)* – 0.0148**Stimulated (1-hour after MMTT) blood glucose level (mg/dL)* – 0.0133* *Stimulated (2-hour after MMTT) blood glucose level (mg/dL)* + 0.250**Beta score 3 months after TP-IAT* – 0.0501**BETA-2 score 3 months after TP-IAT*

Prediction of Goal HbA1c Control at 1 Year: Logistic regression formula using all predictors *P* < .05 in random forests analyses

In the formula below, π indicates the probability of meeting goal HbA1c control 1 year after TP-IAT.

logit(π) = 17.9 – 2.61**HbA1c level 3 months after TP-IAT (%)* – 0.00248**Fasting blood glucose level (mg/dL)* – 0.0138**Stimulated (1-hour after MMTT) blood glucose level (mg/dL)* – 0.0022* *Stimulated (2-hour after MMTT) blood glucose level (mg/dL)* + 0.116**BETA-2 score 3 months after TP-IAT* + 0.0527**SUITO index 3 months after TP-IAT* – 0.0304**HOMA2-%B 3 months after TP-IAT*

Prediction of Insulin Independence at 1 Year: Simple logistic regression formula using only transplanted islet dose and BETA-2 score

In the formula below, π indicates the probability of being insulin independent 1 year after TP-IAT.

logit(π) = -4.14 + 0.282**Transplanted islet dose (1000 IEQ/kg)* + 0.172**BETA-2 score 3 months after TP-IAT*

Prediction of Goal HbA1c Control at 1 Year: Simple logistic regression formula using only transplanted islet dose and BETA-2 score

In the formula below, π indicates the probability of being insulin independent 1 year after TP-IAT.

logit(π) = -3.07 + 0.185**Transplanted islet dose (1000 IEQ/kg)* + 0.297**BETA-2 score 3 months after TP-I*
